# Activity of Novel Ultrashort Cyclic Lipopeptides against Biofilm of *Candida albicans* Isolated from VVC in the Ex Vivo Animal Vaginal Model and BioFlux Biofilm Model—A Pilot Study

**DOI:** 10.3390/ijms232214453

**Published:** 2022-11-21

**Authors:** Paulina Czechowicz, Joanna Nowicka, Damian Neubauer, Grzegorz Chodaczek, Paweł Krzyżek, Grażyna Gościniak

**Affiliations:** 1 Department of Microbiology, Faculty of Medicine, Wrocław Medical University, 50-368 Wrocław, Poland; 2 Department of Inorganic Chemistry, Faculty of Pharmacy, Medical University of Gdańsk, 80-416 Gdańsk, Poland; 3 Bioimaging Laboratory, Łukasiewicz Research Network–PORT Polish Center for Technology Development, 54-066 Wrocław, Poland

**Keywords:** *Candida*, biofilm, BioFlux, ex vivo model, ultrashort cyclic lipopeptides, vulvovaginal candidiasis

## Abstract

In recent years, clinicians and doctors have become increasingly interested in fungal infections, including those affecting the mucous membranes. Vulvovaginal candidiasis (VVC) is no exception. The etiology of this infection remains unexplained to this day, as well as the role and significance of asymptomatic vaginal *Candida* colonization. There are also indications that in the case of VVC, standard methods of determining drug susceptibility to antifungal drugs may not have a real impact on their clinical effectiveness—which would explain, among other things, treatment failures and relapse rates. The aim of the study was to verify the promising results obtained previously in vitro using standard methods, in a newly developed ex vivo model, using tissue fragments of the mouse vagina. The main goal of the study was to determine whether the selected ultrashort cyclic lipopeptides (USCLs) and their combinations with fluconazole at specific concentrations are equally effective against *Candida* forming a biofilm directly on the surface of the vaginal epithelium. In addition, the verification was also performed with the use of another model for the study of microorganisms (biofilms) in vitro—the BioFlux system, under microfluidic conditions. The obtained results indicate the ineffectiveness of the tested substances ex vivo at concentrations eradicating biofilm in vitro. Nevertheless, the relatively most favorable and promising results were still obtained in the case of combination therapy—a combination of low concentrations of lipopeptides (mainly linear analogs) with mycostatic fluconazole. Additionally, using BioFlux, it was not possible to confirm the previously obtained results. However, an inhibiting effect of the tested lipopeptides on the development of biofilm under microfluidic conditions was demonstrated. There is an incompatibility between the classic in vitro methods, the newer BioFlux method of biofilm testing, offering many advantages postulated elsewhere, and the ex vivo method. This incompatibility is another argument for the need, on the one hand, to intensify research on the pathomechanism of VVC, and, on the other hand, to verify and maybe modify the standard methods used in the determination of *Candida* susceptibility.

## 1. Introduction

Vaginal infections, including vulvovaginal candidiasis (VVC), have gained increased relevance in everyday medical practice, which is also reflected in the scientific literature. Being the second most commonly diagnosed gynecological infection, VVC affects over 75% of women of reproductive age worldwide and is often associated with therapeutic failures and the risk of recurrence. The most common and relatively easily recognized etiological factors of this type of infection are yeast-like fungi of the genus *Candida*. Physicians and scientists have been paying more and more attention to mucosal candidiasis—including vulvovaginal candidiasis. Since it is not an infection that requires hospitalization, and its symptoms, as well as possible complications and relapses, are classified as relatively mild, it can be easily underestimated. In recent years, there has been a growing interest in VVC among clinicians and scientists, as well as among patients themselves [1,2,3,4,5,6]. However, despite the increasing amount of research conducted on the pathogenesis of VVC and its relapsed form, as well as on effective treatment, it turns out that more remains unknown than known. Given the difficulties associated with establishing the detailed mechanism of the development of fungal infection on the vaginal mucosa, it becomes virtually impossible to identify a specific factor as a target for potential new antimicrobials [6,7,8,9,10]. It is, therefore, not surprising that, on the one hand, intensive research is being carried out to identify the specific pathogenesis of these infections (which would facilitate the fight against and prevention of these infections [2,3,7]), and on the other hand, many research studies have focused on the search for new substances or therapeutic regimens with potential antifungal activity in the vagina. Because it has been known for years that *Candida* biofilm plays a role (if not the key role) in treatment resistance and VVC relapses, the newly synthesized agents are also tested for anti-biofilm activity [2,3,7,8,11,12,13].

Short cationic compounds are probably the largest group among the compounds that can be an alternative to mycobiotics conventionally used against yeast-like fungi. Among them, ultrashort cationic lipopeptides (USCLs) deserve special attention, as they are especially active against fungi. Their structure consists of short positively charged peptides (seven amino acid residues at most), mainly due to lysine (K) or arginine (R) residues, linked to a fatty acid chain [14,15,16,17]. As a result, these compounds are amphiphilic and easily react with the negatively charged components of the *Candida* cell membrane—sialic acid and phosphatidylinositol [18,19]. All of this results in the permeabilization of these membranes and, in effect, cell death [15,16,17,20]. In addition to antifungal activity, USCLs also show many other properties as antimicrobial and anti-adhesive activity, translating to their potent anti-biofilm effect [21].

In recent years, commonly used laboratory methods to determine the effect of such compounds have been considered a significant obstacle in the research on new antifungal and antibiofilm agents. Researchers often point out that in vitro methods, which primarily determine the MIC (minimum inhibitory concentration) and MBEC (minimum biofilm eradication concentration) values, may turn out to be inadequate in relation to the actual in vivo activity of the substance. Hence, various models are increasingly used, including in vivo and ex vivo models, which are mainly animal (mouse and rat) models [12,22]. So far, the uniqueness of the vaginal microenvironment and the crucial importance of the as yet unexplained (but definitely existing) interactions between *Candida* and vaginal epithelium cells (VECs) have been proven. While there are already relatively well-described models of mucosal candidiasis in the oral cavity or the gastrointestinal tract in research, a reliable and universal research model for VVC remains a challenge [12,22]. In 2010, Harriot et al. were the first to obtain *Candida* biofilm on the surface of vaginal tissue in in vivo and ex vivo animal models. Their comparison showed that in both types of models, the kinetics of biofilm formation and its structure are the same and could be used in further research, as well as that they most likely correspond to biofilms obtained in vitro, e.g., on silicone disks [12]. In 2016, Krom and Willems also compared other methods of testing *Candida* biofilm and its drug susceptibility in vitro, indicating an interesting method for studying this structure under dynamic and realistic microfluidic conditions using the BioFlux-based system [23]. The BioFlux system has many advantages, including the most important two: observing the behavior of microorganisms (biofilms) under microfluidic conditions, thanks to the performance of all experiments in the microcapillaries of this system and the possibility of real-time observation of the processes taking place. The study of fungal biofilm in the flow may have some practical impact depending on the site of infection—it is obvious that this model will be much more useful for imitating conditions prevailing in, for example, blood vessels than in mucosal infections. Referring to the strains isolated from VVC, BioFlux enables continuous 24-hour observation of the kinetics of biofilm development, including *Candida* adhesion and germination, the production of germ tubes and filaments, as well as the subsequent detachment of fragments of the mature structure and its “migration” further. The advantage of BioFlux over standard in vitro methods in drug susceptibility testing is manifested in the assessment of the results—the possibility of observing and assessing the formation of microbial aggregates, their developmental form, and increasing or decreasing coverage of microcapillaries—without the need to take into account potential errors and problems resulting, for example, from manual handling of microscope slides or rinsing of the biofilm. Apart from the evaluation of dynamic processes, BioFlux also enables molecular mechanisms, and in the adjacent microcapillaries, one can simultaneously conduct many other experiments [23,24,25].

In our previous work, we compared the antifungal activity of two pairs of cationic lipopeptides against *Candida* strains isolated from vulvovaginal candidiasis. These compounds effectively eradicated the mature structure of the biofilm [26]. In in vitro studies using polystyrene plates, we have demonstrated the advantage of the newly synthesized cyclic analogs C_16_-CKKKKC-NH_2_ and C_16_-CKRKKC-NH_2_ over their linear counterparts in the antimicrobial activity against *Candida* in planktonic and biofilm form [26]. At the same time, we have already proven that the use of combination therapy in the form of the simultaneous action of antifungal fluconazole that is conventionally used in VVC and the tested lipopeptides could be equally promising—this time indicating the advantage of the linear compounds C_16_-KKKK-NH_2_ and C_16_-KRKK-NH_2_ [26]. As we have mentioned before, such an approach could solve the problem of the relatively high toxicity of this type of compound in relation to eukaryotic cells, because the combination of USCLs with fluconazole turned out to be effective at concentrations several times lower than when these substances were used separately [26].

The aim of the study was to verify the results obtained previously in classic in vitro tests using the ex vivo VVC mouse model, and in the in vitro biofilm model under microfluidic conditions with the BioFlux-based system. The most promising pair of lipopeptides selected on the basis of previous work were used: linear C_16_-KKKK-NH_2_ and cyclic C_16_-CKKKKC-NH_2_. Their effect on mature fungal biofilm was tested against two selected clinical strains of *Candida* albicans isolated from VVC, for which the drug susceptibility of the biofilm was previously determined. Using effective in vitro concentrations, the activity of the tested lipopeptides was verified both directly on the mouse vaginal tissue (ex vivo model) and under microfluidic conditions (BioFlux model).

## 2. Results

### 2.1. Ex Vivo Animal Model

In all cases, it was possible to obtain 24-h *Candida* biofilm on the surface of the mouse vaginal tissue for both tested isolates. This was confirmed by the results obtained during the homogenization of tissues, as well as by the microscopic photos showing the characteristic long filaments and blastospores of *C. albicans*. The mean CFU per g of tissue for biofilm unexposed to any compounds (positive controls) was 1.93 ± 1.37 × 10^8^ for the CA1 strain and 2.02 ± 1.21 × 10^8^ for the CA2 strain.

Because our previous studies have shown that fluconazole is not effective in the eradication of *Candida* biofilms [26], high concentrations of amphotericin B (50 µg/mL) were used to control the eradication of this structure from tissues. This compound is not used in the treatment of VVC and shows relatively high toxicity; hence, its use was only intended as a control in our ex vivo model. The mean CFU per g for CA1 treated with AMB was 1.03 ± 1.11 × 10^7^ and 1.08 × 10^7^ ± 7.91 × 10^6^ for CA2. This results were statistically significant (*p* < 0.05), which can be clearly seen in the Supplementary Materials in Figures S1–S4.

**Figure 1:**
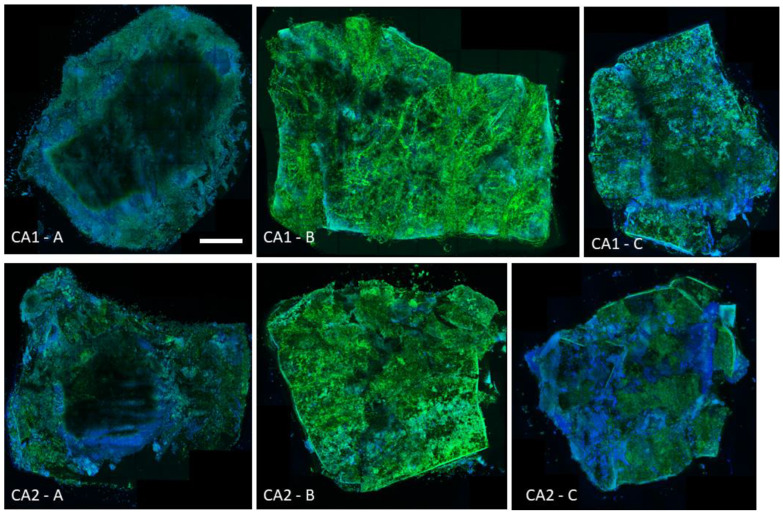
Mature 24-h biofilm of *Candida* albicans strains CA1 and CA2 isolated from VVC on mouse vaginal epithelium tissue (ex vivo model) under the confocal microscopy. Green color shows *C. albicans* based on Calcofluor staining and blue labels the vaginal epithelium based on Evans blue dye. CA**1–A** and CA**2–A**—negative controls (tissue without *Candida*); CA**1–B** and CA**2–B**—biofilm of strain number 1 and strain number 2, respectively; CA**1–C** and CA**2–C**—biofilms treated with amphotericin B (50 µg/mL). Scale bar = 1 mm.

Representative microscopic images corresponding to the described results are shown in Figure 1.

**Figure 2:**
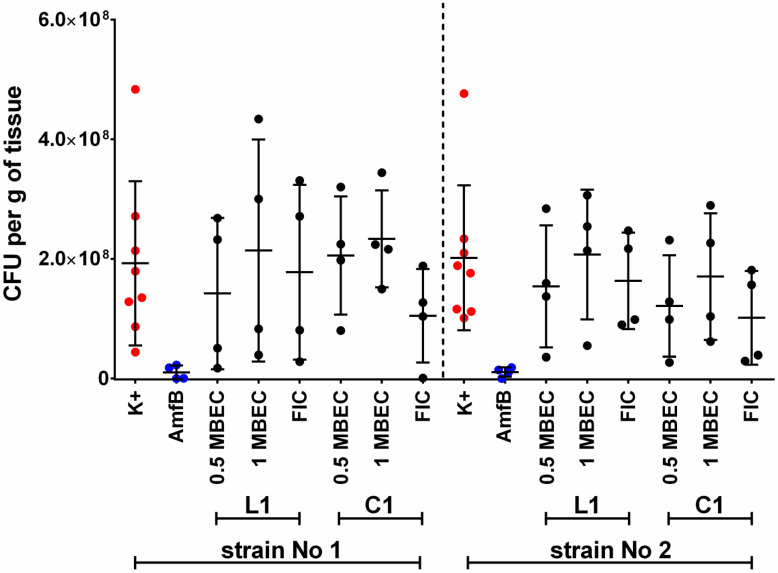
Distribution of CFU per gram of tissue values obtained for strains CA1 and CA2 in all courses of experiments. K(+) is referred to as a positive control (untreated biofilm of *C. albicans*). Biofilms were treated with: AMB—amphotericin B (50 µg/mL), lipopeptides L1 and C1 at a concentration equal to MBEC, ½ MBEC, and FIC values (MBEC—minimum biofilm eradication concentration; FIC—fractional inhibitory concentration).

The experiments showed that neither of the tested L1 and C1 lipopeptides is effective in eradicating *C. albicans* biofilm from the surface of the vaginal tissue of mice at concentrations equal to MBECs and 1/2 MBECs (Figure 2).

In the case of eradicating concentrations (MBECs) for the linear L1 compound, the average was 2.14 ± 1.86 × 10^8^ and 2.07 ± 1.09 × 10^8^ CFU/g of tissue for CA1 and CA2, respectively. Similar values were obtained for the C1 cyclic lipopeptide. For CA1, CFU/g of tissue averaged 2.33 × 10^8^ ± 8.11 × 10^7^, and for CA2, the mean CFU/g was 1.70 ± 1.06 × 10^8^, which would also indicate a decrease relative to the biofilm not exposed to any compounds. Statistical analysis showed no significance (*p* > 0.05) in the described results (Figures S1–S4 in Supplementary Materials).

When sub-inhibitory concentrations of lipopeptides (1/2 MBECs) were used, the results were slightly more optimistic. For USCL L1, the mean values of obtained CFU/g indicated the eradicating effect of this lipopeptide for both strains—1.42 ± 1.26 × 10^8^ for CA1 and 1.54 ± 1.02 × 10^8^ for CA2. The eradicating effect of the C1 analog was indicated by the mean CFU/g tissue result obtained in the case of CA2–1.21 × 10^8^ ± 8.48 × 10^7^. For strain CA1, the mean value was 2.05 × 10^8^ ± 9.88 × 10^7^ CFU/g. In this case, the described differences also turned out to be statistically insignificant (*p* > 0.05) (Figures S1–S4 in Supplementary Materials).

Compound L1 with FLC shows that the use of combinations of fluconazole with the tested lipopeptides, at concentrations equal to FICs, eradicated the *C. albicans* biofilm of both strains used (Figure 2). For compound L1 with FLC, a decrease in CFU/g to 1.78 ± 1.46 × 10^8^ and 1.63 × 10^8^ ± 8.07 × 10^7^ for CA1 and CA2, respectively, was obtained. Similarly, in the case of the cyclic C1 analog with FLC for strain CA1, the CFU/g value was 1.05 × 10^8^ ± 7.80 × 10^7^ and 1.01 × 10^8^ ± 7.84 × 10^7^ for CA2. However, again, statistically significant differences between the obtained results could not be proven (*p* > 0.05) (Figures S1–S4 in Supplementary Materials).

For the negative controls (tissues without any strain suspension) no growth was observed in all courses of the experiments (CFU/g was 0).

The described results combined together in one graph with standard deviations are shown in Figure 2.

**Figure 3:**
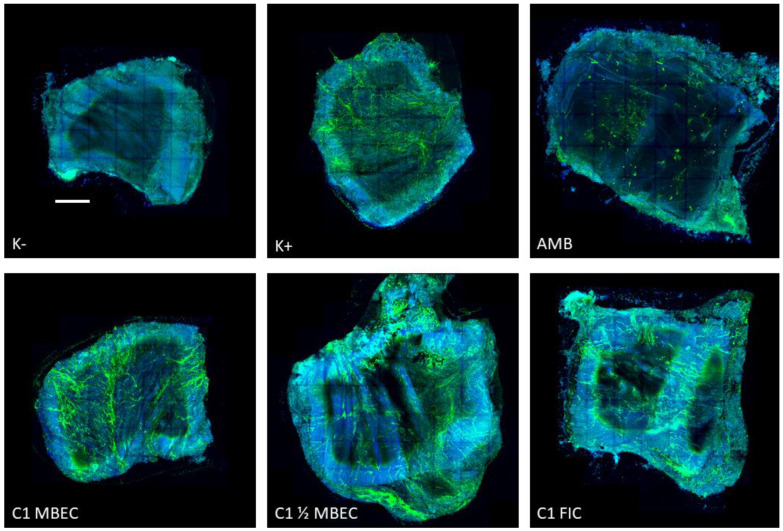
An exemplary set of experiments in confocal microscopy. Green color shows *C. albicans* based on Calcofluor staining, and blue labels the vaginal epithelium based on Evans Blue dye. Mature biofilm of *C. albicans* CA2 treated with: AMB—amphotericin B (50 µg/mL) and cyclic lipopeptide C1 at a concentration equal to MBEC, ½ MBEC, and FIC. K(−) and K(+) are referred to as negative and positive controls (MBEC—minimum biofilm eradication concentration; FIC—fractional inhibitory concentration). Scale bar = 1 mm.

A representative set of experiments in confocal microscopy is presented in Figure 3. As there is always a risk of poorly reflecting quantitative results (such as CFU/g) in microscopy when presenting fragments of photos, it was decided to use a technique that allows for microscopic mapping of the whole of the analyzed tissue. As a result, the overall coverage of the epithelium by the *Candida* biofilm, and especially certain areas of greater filament densities, can be seen in the attached figures. It can also be seen that the surface of this tissue is not completely flat. When comparing the quantitative values with images from confocal microscopy, it is worth noting that the application (according to the methodology of Harriot et al.) of the Calcofluor dye, along with Evans blue, also stains tissue cells (blue color), not only fungal cells (green color). Importantly, however, the significant quantitative differences, expressed as CFU/g and calculated in Figure 2, are clearly visible in Figure 3 as the difference between the negative and positive controls and the eradicating effect of amphotericin B. More subtle differences in CFU/g, which are described above, are virtually impossible to observe in CM, and the amount of green-stained *Candida* biofilm filaments on tissues treated with lipopeptide in the concentrations of MBEC, ½ MBEC, and FIC is very difficult to compare on the basis of microscopic observation alone.

### 2.2. BioFlux Biofilm Model

**Figure 4:**
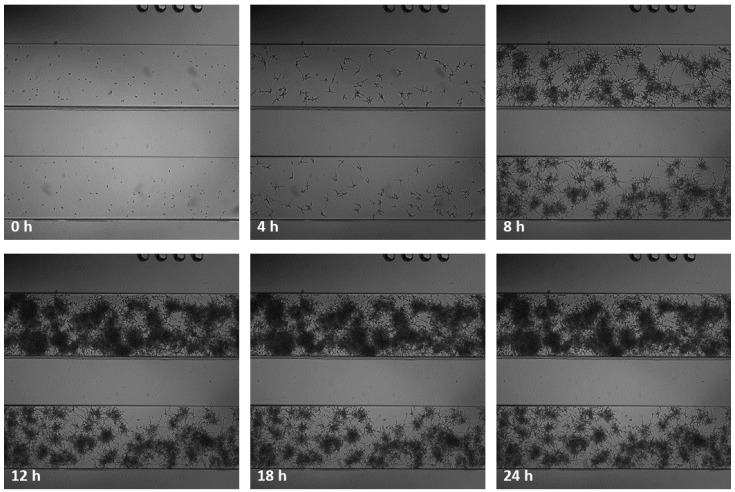
Biofilm formation of *C. albicans* strain CA1 in two separate channels (biofilm formation phase), in a time-lapse series taking images every 1 h over 24 h of incubation in microfluidic conditions with RPMI 1640 medium (without additional compounds), using BioFlux Z1000 system. The presented photographs were taken at the start of the experiment (0 h), and after 4, 8, 12, 18, and 24 h.

By applying the BioFlux equipment, it was also possible to obtain mature, 24-h biofilms in microfluidic conditions for both *Candida* strains. Thanks to the possibility of using real-time monitoring of the experiments, the time-lapse series microscopic photos were taken every 1 h for 24 h. These photos clearly show the presence of *Candida* blastospores, which, after 2 h of incubation in RPMI 1640, begin to form germ tubes and then long filaments. From hour 10 to hour 11 of incubation, no further changes in the created biofilm structure could be captured in the photographs. Representative images of biofilm formation over time for strain number 1 are shown in Figure 4. In Supplementary Materials, Videos S1 and S2 provide animations of all time-lapse microscopic shots captured over the 24 h of incubation of strain CA1 and CA2, respectively.

In the course of experiments using the BioFlux Z1000 system, the influence of both tested lipopeptides L1 and C1 at concentrations equal to MBECs was analyzed. By comparing the average percentage of increase in biofilm biomass over 24 h of incubation, the inhibitory effect of both compounds in relation to the control samples was visible, although the observed differences turned out to be statistically insignificant (*p* > 0.05). What can be noted is that the influence of the linear L1 analog was found to be the most favorable, especially for strain CA2—in the 8th hour of incubation, the increase was 0.29%, while in the control, it was 2.45%. The final (24 h) increase in biofilm biomass of CA2 for this USCL was slightly more than a half of the value obtained in the control (4.35% vs. 8.60%). The analogous gain for CA1 was 6.88% in the presence of compound L1, compared to 9.39% for the control. The inhibitory effect of the cyclic C1 analog was also seen for both strains and, again, a more favorable result was obtained with CA2. The increase for this strain was 6.18% in the presence of C1 vs. 8.60% for the untreated biofilm. For CA1, the difference in biofilm growth observed was smaller, whereas in the control, it was 9.39%. Treatment with compound C1 lowered this value to 8.40% of channel coverage. On the other hand, for CA1, the increase in channel coverage in the presence of C1 was slightly smaller than in the positive control (8.40% vs. 9.39%). Nevertheless, according to the statistical analysis, the eradicating effect of the tested USCLs at concentrations equal to MBEC against mature *Candida* biofilm could not be obtained in the Bioflux biofilm model.

**Figure 5:**
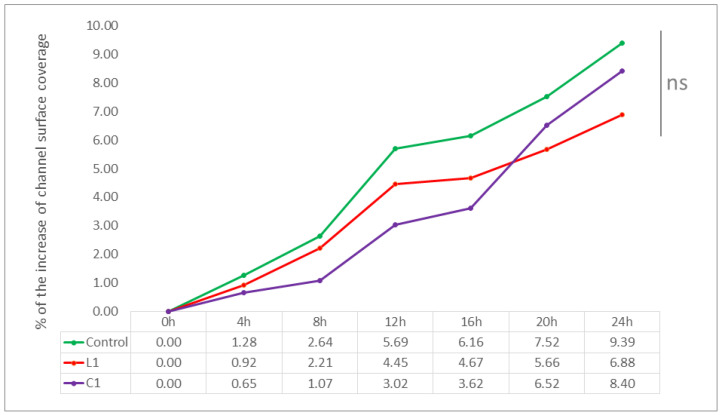
Growth dynamics of biofilm biomass of *C. albicans* strain CA1 over 24 h in the presence of lipopeptides L1 and C1, expressed as a percentage of channel coverage in the BioFlux Z1000 system. For all tested samples, the biofilm obtained after 24 h of growth in the absence of any substances (a biofilm development phase) was taken as the starting point. All experiments were repeated in triplicate. NS—non-significant.

**Figure 6:**
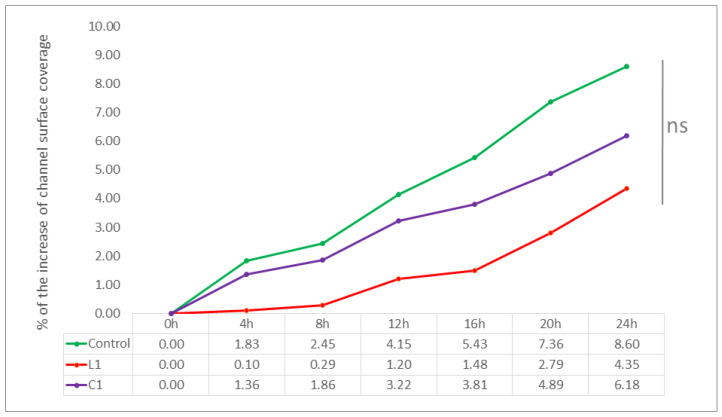
Growth dynamics of biofilm biomass of *C. albicans* CA2 over 24 h in the presence of lipopeptides L1 and C1, expressed as a percentage of channel coverage in the BioFlux Z1000 system. For all tested samples, the biofilm obtained after 24 h of growth in the absence of any substances (a biofilm development phase) was taken as the starting point. All experiments were repeated in triplicate. NS—non-significant.

The reported results, together with the exact values obtained at certain time points, are shown in Figure 5 for CA1 and in Figure 6 for CA2.

**Figure 7:**
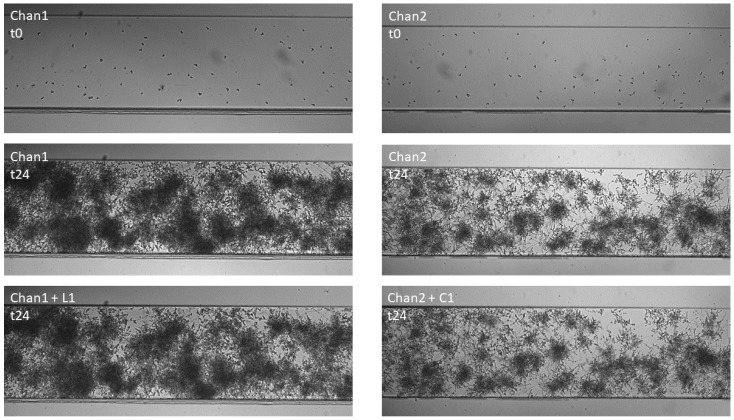
*C. albicans* CA1 biofilm formation and eradication, incubated in RPMI1640 medium under microfluidic conditions, in two separate channels (Chan1 and Chan2) of the BioFlux Z1000 system. t0—start of experiments, CA1 blastospores; t24—formed 24-h mature CA1 biofilm; L1/C1 t24—24-h eradication of CA1 biofilm with lipopeptides L1 or C1, respectively.

Exemplary set of microscopic images of the CA1 strain biofilm formation phase (first 24 h) and eradication phase (next 24 h), with L1 and C1 visible in Figure 7 below.

## 3. Discussion

Lipopeptides are a group of substances with a wide range of antimicrobial activity already known to researchers. In our previous work, using classical in vitro methods (determination of MIC, MBEC, and FIC values), we proved the effectiveness of the newly synthesized ultra-short cyclic lipopeptides (USCLs) against yeast-like fungi, both in planktonic and biofilm form [15,26]. Among other properties, USCLs have recognized anti-adhesive properties and, thus, anti-biofilm and antimicrobial action, including against *Candida* [14,15,16,17]. For this reason, we are investigating the effect of these compounds on *Candida* strains isolated from vulvovaginal candidiasis (VVC). Both in the scientific literature and in everyday clinical practice, attention is increasingly being paid to this infection for several reasons, including its extremely common occurrence, the still unknown etiology, and the increasingly frequent and difficult to explain treatment failures and recurrences of VVC. Based on our previous research, two (linear and cyclic) of the most promising USCLs were selected for further study. Their structure consists of a palmitic acid residue (C16, hexadecenoic acid) conjugated to a positively charged peptide with L-lysine residues and C-terminal amide. In the case of the cyclic analog, cyclization was achieved with two cysteine (C-cysteine) residues linked by a disulfide bridge [16,19]. In our study, the compounds C_16_-KKKK-NH_2_ (L1) and C_16_-CKKKKC-NH_2_ (C1) were able to inhibit the plankton growth of various *Candida* strains isolated from VVC, as well as to eradicate the mature biofilm structure formed by these isolates at lower concentrations than the other tested USCLs [26]. At the same time, they showed relatively low toxicity towards HaCaT and the lowest hemolytic capacity [15]. In addition, they showed the most favorable (synergistic or additive) effect when using combination therapy—the simultaneous use of these lipopeptides in combination with antifungal fluconazole, which is conventionally used in VVC [26].

The aim of this study was to verify the results obtained previously using standard in vitro methods for determining drug susceptibility. This was performed using two other methods of testing the effect of various compounds on *Candida* biofilm—a newly developed ex vivo model, in which we used mouse vaginal tissues, and a biofilm model in microfluidic conditions, obtained by using BioFlux technology. Two *C. albicans* strains isolated from VVC were selected for the study, with MIC, MBEC, and FIC values previously determined for fluconazole as well as for L1 and C1 lipopeptides [26]. The experiments consisted of creating a mature (i.e., 24-h) biofilm using these strains, and then treating this structure with the tested compounds at concentrations considered in the course of previous studies to be the lowest which were effective in eradication. The expected result was at least a notable reduction in biofilm biomass under the influence of L1 and C1 (at concentrations equal to MBEC) and their combination with fluconazole (at concentrations equal to FIC), similarly to the RPMI 1640 microdilution method on polystyrene plates. In addition, it was decided to use sub-inhibitory concentrations of lipopeptides in the model using mouse vaginal tissues. There exist reports on the potential action of certain AMPs (antimicrobial peptides), including cationic lipopeptides, resulting in sensitization of various microorganisms to the effects of the so-called host defense peptides (HDPs) which naturally occur in the body. Although the mechanism of this action is currently unknown, it has already been observed that the addition of low (sub-inhibitory) concentrations of AMPs to, for example, serum may inhibit bacterial growth, most likely enhancing the action of complement proteins, lysozyme, or lactoferrin [16,27,28]. It is known that there is a mucus layer on the vaginal mucosa and in the vagina itself, produced by the VEC, which has antimicrobial properties and contains many substances, including lactoferrin and lysozyme [29,30]. Unfortunately, in vitro, it is almost impossible, or at least extremely difficult, to recreate or obtain this substance in a manner similar to, for example, the collection of saliva or serum. In addition, obtaining a tissue culture of VK2 cells would not reflect the complex mechanisms between VECs, their products, or yeast-like fungi. Hence, it was decided that we would test the hypothesis of the potential supporting action of lipopeptides at sub-inhibitory concentrations against various, including unidentified, HDPs in the vaginal microenvironment using an ex vivo animal model.

The MBECs were identical for both tested *C. albicans*. In the in vitro microdilution method in RPMI 1640 on polystyrene plates, the use of these concentrations resulted in no signal (MTT solution color change) when visualizing the results, which meant that no metabolically active sessile cells were present [26]. In an ex vivo animal model using mouse vaginal tissue fragments, treatment of the 24 h biofilm formed by these strains with the same concentrations of L1 and C1 did not eradicate *Candida*. In confocal microscopy, a thick layer of fungal hyphae stained with Calcofluor White was still visible. The failure of biofilm eradication in this model was demonstrated by the results obtained with tissue homogenization as well. For both the CA1 and CA2 strains, in the presence of L1 equal to MBEC, the mean values of CFU/g of tissue were generally higher than in positive controls—Figure 2 and Figures S1 and S3 in Supplementary Materials; no statistical significance (*p* > 0.05). For strain CA1, when using C1, the value of CFU/g visibly exceeded the result for the positive control. A low decrease in mean value was only obtained for CA2 using C1 (again, without statistical significance). However, it should be kept in mind that the obtained results may be subject to some variations depending on various laboratory factors. Examples of such steps potentially leading to greater uncertainty include manual homogenization of tissue fragments or transfer of tissues with strain suspensions to microscope slides in CM. Furthermore, one should remember the tissue-dependent characteristics, as the tissues were obtained from successive mice. Therefore, the noticeable standard deviations shown in Figure 2 should not be surprising either. Nevertheless, the presence of a relatively large amount of *Candida* (most often exceeding the values obtained in positive controls) is certain, despite the use of eradicating lipopeptide concentrations. In no case was there a decrease in the amount of fungi on the surface of the tissue, which was comparable, for example, to the significantly lower values obtained for high concentrations of amphotericin B (Figure 2, Figures S1–S4 in Supplementary Materials) (*p* < 0.05). On this basis, it can be concluded that the results obtained with the classical in vitro methods were not confirmed in the ex vivo method. Of course, the question remains as to which of the methods is more reliable. Microdilution methods have been recognized and approved for many years as reliable by all major institutions and scientific and clinical societies; they are repeatable and verifiable. Their translation into the clinical use of the tested compounds is also confirmed and effective [31,32]. However, there are some clinical exceptions, and we believe that vulvovaginal candidiasis should be considered such an exception. This is because, despite establishing the drug susceptibility of *Candida* isolates in VVC therapy, failures still occur [2,3,6,8]. The discussion on what we actually know about the biofilm formed by microorganisms and what the structure which we clinically consider biofilm, and above all, in vitro, really is, has been going on for several years [33]. When considering the interpretation of the results of our work, these issues are slightly less important—it does not seem to matter whether the 24-h structure formed by the suspension of *C. albicans* plankton cells on the surface of the tissue is called biofilm. In the ex vivo model, we have the opportunity to observe how yeast-like fungi behave towards the vaginal epithelium, as well as how various compounds, including the USCLs tested, may act in this environment. In 2010, Harriot et al. conducted a comparative analysis of the *Candida* biofilm itself, and of the kinetics of its formation, in the in vivo, ex vivo model as well as on polystyrene plates in RPMI 1640. They concluded that the dynamics of biofilm formation and its individual stages, as well as the structure and properties of mature (24-h) biofilm, are comparable and almost identical [12]. If so, the reason for the differences in the results of biofilm eradication by L1 and C1 lipopeptides obtained with these methods should be seen in the interaction of fungi with the abiotic and biotic surfaces. On the surface of the vaginal tissue, *Candida* undoubtedly interact with epithelial cells, deriving primarily nutrients from them (no nutrient medium for fungi is used in the ex vivo model) [34]. According to the current knowledge, *C. albicans* has the ability to actively invade tissues thanks to many virulence factors, including filamentation. Thus, the hyphae observed on the epithelium should be considered an invasive form of these strains, displaying, at the same time, tissue-destructive activity [29,35]. It is also known that the proliferating and filamentous *Candida* blastospores also have the ability to produce a large amount of extracellular substance, or mucus (matrix–ECM in accordance with the dynamics of biofilm formation), which is a barrier impermeable to various substances [6,8,12]. On the surface of a polystyrene plate, an abiotic material, yeast-like fungi also multiply intensively and have the ability to germinate and produce mucus. However, this method provides a nutrient medium—RPMI 1640—so these isolates do not have to compete for substances, and have no possibility of further invasion. Perhaps this is the reason why it is “easier” to eradicate yeast-like fungus overnight on a given surface (which we call mature biofilm) in an in vitro model than in an ex vivo tissue model. If so, the method that uses the animal equivalent of the vaginal epithelium, which undoubtedly more closely imitates the real processes of VVC, seems to be much more reliable than the polystyrene plates. If, in the course of further studies, the described observations and results are confirmed, it could indicate the need to drastically change the methods of *Candida* susceptibility testing used in microbiological diagnostics towards VVC.

The use of concentrations corresponding to the lowest FIC also did not result in complete eradication of *C. albicans* biofilm for either tested strain. However, the use of FIC resulted in the lowest mean number of CFU/g of tissue for the entire experiment (no statistical significance, *p* > 0.05). A decrease can be seen for CA1 and for CA2, for both lipopeptide–fluconazole combinations (Figure 2, Figures S1–S4 in Supplementary Materials). It is worth emphasizing that the fact that the classic checkerboard method used by our team in the previous work to determine the FIC and FIC indexes refers to plankton cells and not to biofilm [26] speaks in favor of the presented results. Thus, naturally, one would expect higher MICs of both substances in combination during eradication of this highly resistant structure than against *Candida* plankton. Meanwhile, concentrations many times lower than MBEC (and MIC) turned out to be partially effective in our ex vivo method. A comparison of the results obtained in our work (Table 1) suggests that the simultaneous use of linear USCL concentrations 16 times (isolate 1) and 32 times (isolate 2) lower (than MBEC), as well as the use of concentrations of cyclic USCL 32 times (both isolates) lower (than MBEC), with the addition of fluconazole at a concentration 64 times lower than the MIC, produces similar or even better results in eradication of *C. albicans* biofilm from the surface of the murine vaginal epithelium [26]. The above may also prove that the combination of substances with different mechanisms of action can actually be more effective, which has been postulated for a long time [36,37,38,39]. As we mentioned in our previous work, the most likely explanation of the activity of these compounds against *C. albicans* is the possibility of sensitizing fungal cells by means of USCLs, which is conducted by causing at least partial damage to cell membranes, enabling faster and more effective interaction of fluconazole with its molecular target inside the cell (14 α-ergosterol demetylase). At the same time, the very interaction of this azole with the cell membrane may increase its permeabilization by lipopeptides [26]. Proving the effectiveness of using a combination of lipopeptides with fluconazole at concentrations many times lower than, when these substances are used separately, could solve the two most serious problems that should be taken into account in research on antimicrobial activity. The use of lipopeptides at much lower concentrations could no longer be associated with the potential relatively high toxicity of these compounds, which is increasingly noted. In addition, by treating cells with substances with different mechanisms of action, the acquisition of resistance by microorganisms, including *Candida*, would be significantly hampered, especially since the acquisition of resistance to AMPs, including USCLs, is already considered unlikely [16]. Thus, the results obtained in the course of the presented studies on the eradication of *C. albicans* biofilm from the tissue, using a combination of low concentrations of fluconazole and both lipopeptide analogs, are highly promising and require further confirmation.

It seems that the most diverse and difficult to interpret results of tissue biofilm eradication were obtained using sub-inhibitory concentrations (1/2 MBEC). Results were, again, statistically insignificant (*p* > 0.05). With the use of L1, some reduction in mean CFU/g values was obtained for both *C. albicans* strains (Figure 2, Figures S1 and S3 in Supplementary Materials). Regarding C1, one value indicates an eradicating effect—a decrease in mean CFU/g for CA2. For CA1, the obtained values exceeded those determined for the positive controls. It can, therefore, be concluded that the sub-inhibitory MBEC concentrations of the linear lipopeptide have a similar eradicating capacity to the use of the combination of this analog with fluconazole. This would be in contrast to the cyclic USCL, which is actually ineffective at one-half of the MBEC concentrations. At present, it is difficult to determine why a more favorable effect of linear analogs at low concentrations could be observed, since when using the lipopeptide alone, cyclic compounds turn out to be much more effective against *Candida* (and their MIC values are much lower). It is worth noting, however, that both lipopeptides showed synergistic or additive activity with fluconazole, and their combinations were comparably effective in eradicating fungal biofilm from the tissue. It is likely that, in some way, a linear analog at low concentrations sensitizes *Candida* cells to the effects of fluconazole when used in combination therewith, and also analogously enhances the antifungal activity of HDPs and other immune mechanisms present in the vaginal tissue [16,27,28]. If the cyclic compound acts similarly in combination with fluconazole, and if it is indeed possible, for example, to synergize with HDPs, why does C1 not have this ability when L1 does? Any potential explanation must take into account the differences in the structure of both compounds, as well as their interactions with VECs and the cells of the immune system, and with the substances they produce. Even if the aforementioned hypothesis regarding supporting the action of HDPs and/or other immunological components is true, its confirmation requires many verification studies, focused primarily on determining the nature of the interaction of all the substances and components present in the vagina—especially in the presence of *Candida* fungi.

Under the microfluidic conditions obtained using the BioFlux technology, it was possible to obtain a 24-h structure formed by blastospores and hyphae of both tested *C. albicans* strains—which we call biofilm. The microscopic photos of the channels used in BioFlux, taken every hour, clearly showed the next stages of the development of fungal biofilm—especially visible in Videos S1 and S2 animations and in the example in Figure 4. These observations are consistent with the common knowledge on the kinetics of biofilm formation, and (not for the first time) they confirm the equal suitability of the microflow model and in vitro models, as well as of the in vivo and ex vivo animal models, already proven by Harriot et al. [6,13]. BioFlux is currently considered to be another method of testing the biofilm of microorganisms and its drug susceptibility in vitro. Thanks to the use of BioFlux technology, we were able to observe the behavior of the strains in real time. We managed both to confirm the occurrence of all the commonly described stages of fungal biofilm formation, and to visually and quantitatively verify the effect (or lack thereof) of the tested substances on the investigated *C. albicans* isolates. According to the assumptions, if, for example, yeast-like fungi behave similarly/almost identically on polystyrene plates and in BioFlux channels, they should also show at least similar drug sensitivity when exposed to the same eradicating substances [23]. Following these assumptions, we treated the 24-h structure formed by *C. albicans* strains with L1 and C1 lipopeptides at concentrations equal to MBEC—again, expecting to confirm the effectiveness of these compounds in combating biofilm in vitro. Meanwhile, the results obtained by us do not prove the eradication abilities of USCLs, but the slight (and statistically insignificant) possibilities of limiting the further development of fungal biofilm. In Figure 5 and Figure 6 for strains CA1 and CA2, respectively, we can observe lower percentages of increasing coverage of channels in which biofilm was previously formed. Compared to the positive controls, especially for CA2, a relatively markedly lower further increase in the amount of *Candida* on the surface of the channels can be seen. During the first 8 h of incubation with the L1 flow, there was almost no further multiplication of the fungi (value 0.29 vs. 2.45% in K(+)). Ultimately, after one day, the percentage of coverage increase was nearly 50 percent lower than in K(+) (4.35 vs. 8.60%). The analogous results for CA1 also show an insignificant reduction in multiplication only in the first 8 h of incubation with L1 (2.21 vs. 2.64% in K(+)).After 24 h, the final value is lower by almost ¼ (6.88 vs. 9.39% in K(+)). In both cases, compound C1 limited biofilm development of both strains in the channels to a much lesser extent—the final values for CA1 were 8.40% (vs 9.39%), and for CA2, 6.18% (vs 8.60%). In both cases, after 12 h, *C. albicans* formed a very thick layer of biofilm in the channels. This physically, above all, significantly hindered the further flow of any substances. Given the fungal ability to produce a large amount of highly impermeable matrices (ECM), also most likely abundantly present in these cases, the relatively poor penetration of L1 and C1 is not surprising [8,33,40]. It should also come as no surprise that action in the first hours was more effective. It is likely that during the first few flows of substances, there was the greatest mechanical effect on the hyphae present in the channels and on their detachment. These, at the same time, could expose the more sensitive cells located in the deeper layers of the biofilm to the effects of USCLs, limiting their multiplication or even their microbiotic effect (percentages are presented for the entire length of the channels observed under the microscope). The microfluidic model is, perhaps, not the best reflection of *Candida* kinetics and of the interactions with test substances during VVC. However, comparing the results obtained with the use of BioFlux and with polystyrene plates, it can be concluded that for the effective action of lipopeptides against fungal biofilm, constant contact of microorganism cells with antimicrobial substances is necessary, as is the case with polystyrene plates. However, in both models, it is difficult to detect active invasion of *C. albicans* during adhesion to abiotic surfaces, so this in vitro model does not seem to be appropriate for drawing conclusions regarding the behavior of yeast-like fungi on the surface of the vaginal tissue during infection.

## 4. Materials and Methods

### 4.1. Lipopeptide Synthesis

The compounds were obtained by using the method reported previously [15]. Briefly, lipopeptides were synthesized manually by solid-phase Fmoc/tBu methodology. Polystyrene resin modified by Rink Amide linker was used as the solid support (loading ca. 1.0 mmol/g; Orpegen Peptide Chemicals GmbH, Heidelberg, Germany). Deprotection of the Fmoc group was performed with a 20% (*v*/*v*) piperidine (Iris Biotech GmbH, Marktredwitz, Germany) solution in DMF (*N*,*N*-dimethylformamide; POCH, Avantor, Gliwice, Poland) for 15 min. Acylation was conducted with a mixture of DIC:OxymaPure:Fmoc-AA-OH (mole ratio 1:1:1; DIC–*N*,*N*’-Diisopropylcarbodiimide) dissolved in DMF:DCM (1:1, *v*/*v*; DCM—dichloromethane, Chempur, Piekary Slaskie, Poland) in four-fold excess based on the resin for 1.5 h (DIC and OxymaPure; Iris Biotech GmbH, Marktredwitz, Germany). Fmoc-l-Lys(Boc)-OH, Fmoc-l-Cys(Trt)-OH (amino acids were purchased from Orpegen Peptide Chemicals GmbH Heidelberg, Germany), and hexadecanoic acid (C16, palmitic acid; Merck, Darmstadt, Germany) were used in coupling reactions. The peptides were cleaved from the resin using one of the mixtures: (A) TFA (trifluoroacetic acid; Apollo Scientific, Denton, UK), EDT (1,2-ethanedithiol; Merck, Darmstadt, Germany), TIS (triisopropylsilane; Iris Biotech GmbH, Marktredwitz, Germany), and deionized water (92.5:2.5:2.5:2.5, *v*/*v*/*v*/*v*); (B) TFA, TIS, and deionized water (95:2.5:2.5, *v*/*v*/*v*). Mixture A was used with C_16_-CKKKKC-NH_2_ (C1), whereas mixture B was used for C16-KKKK-NH2 (L1). Cleavage was accomplished within 1.5 h under stirring. Then, the peptides were precipitated with cooled diethyl ether (POCH, Avantor, Gliwice, Poland) and lyophilized. The crude peptide with cysteine was dissolved in 20% (*v*/*v*) acetic acid (Chempur, Piekary Slaskie, Poland) solution (0.5 g/L) and oxidized with iodine to obtain the peptide with an intramolecular disulfide bridge. The peptides were purified by RP-HPLC. Pure fractions (>95%, HPLC) were collected and lyophilized. The identity of all compounds was confirmed by mass spectrometry (ESI–MS).

### 4.2. Candida Strains

Microbiological assays were performed on 2 clinical isolates of *Candida albicans* randomly selected from the pool of strains tested in our previous work [26]. Strains (further referred as CA1 and CA2) were originally isolated from the vaginas of women with vulvovaginal candidiasis and were deposited in the Internal Collection of the Department of Microbiology, Wrocław Medical University. The isolates were stored as suspensions in TSB medium enriched with glycerol as described elsewhere, frozen at −80 °C.

The study protocol was approved by the local Bioethics Committee of Wrocław Medical University (No. 774/2018, approval date: 27 December 2018). All experiments were performed in accordance with relevant guidelines and regulations.

### 4.3. Ex Vivo Animal Model

All experiments were carried out on tissues harvested from female C57BL/6 mice, 8–12 weeks of age, purchased from the Jackson Laboratory (Bar Harbor, ME, USA). The mice were housed at the Łukasiewicz Research Network—PORT Polish Center for Technology Development in Wrocław, Poland in individually ventilated cages. A 12:12 h light-dark cycle was established under specific pathogen-free conditions, with water and food available ad libitum. As described by Harriott et al., the mice were euthanized and their vaginae were excised and cut longitudinally to expose the mucosal surface. Each vagina was divided into six sections and placed in a 6-well plate with the mucosal side facing up, in 500 µL of phosphate-buffered saline (PBS) (AppliChem GmBH, Darmstadt, Germany) with penicillin (100 U/mL) and streptomycin (100 μg/mL) to prevent growth of normal vaginal bacteria. Then, *C. albicans* suspensions (subcultured for 24 h on Sabouraud Dextrose Agar with chloramphenicol at a concentration of 100 mg/L) in sterile 0.9% NaCl in concentration of 1–5 × 10^6^ CFU/mL were added and incubated for 24 h at 37 °C with CO_2_ [12]. After this time, fungal biofilms formed on tissues were washed with PBS, and 500 µL of the test compounds was added. Minimum biofilm eradication concentrations (MBECs) of both lipopeptides, as well as fractional inhibitory concentrations (FICs), were determined in our previous study and are presented in Table 1 [26]. Each experiment included a negative control (tissue without *Candida* suspension), positive control (biofilm of *Candida* not exposed to any compound) and four samples treated with the following: high concentration of amphotericin B (50 µg/mL) (referred as AMB), concentration equal to MBEC and ½ MBEC of analyzed lipopeptides, and a combination of lipopeptides with fluconazole equal to FIC value. All samples were prepared in duplicate. Tissues were incubated again for 24 h at 37 °C with CO_2_. One set of the experiment was dedicated to confocal microscopy, and the other to homogenization and quantification of the tissue’s fungal burden. The experiment was repeated four times.

### 4.4. Homogenization of Tissues

All tissues were weighed and homogenized in 1 mL sterile H_2_O using TissueRuptor II (Qiagen, Germantown, MD, USA). Next, 10-fold dilutions in sterile H_2_O were prepared and plated to Sabouraud Dextrose Agar, then incubated overnight at 37 °C. The colonies were counted and CFU per mL and CFU per g (of tissue) values were calculated, as well as mean values and standard deviations.

### 4.5. Microscopy Assay

Vaginal tissues were stained with 1 mg/mL Calcofluor White solution (Fluka) for 20 min at room temperature to visualize yeast and hyphae. Then, the samples were placed with the epithelial side up onto glass microscope slides and covered with a glass coverslip. The slides were examined on a resonant Leica SP8 confocal microscope (Leica Microsystems, Wetzlar, Germany) using a dry 10× objective (NA 0.4). Calcofluor White (labeling *Candida*) was excited with a 405 nm laser line (emission range 410–460 nm), while Evans blue (labeling vaginal epithelium) was simultaneously excited with a 638 nm laser line (emission range 645–720 nm). Whole pieces of tissues were imaged as mosaics, with single tiles being confocal Z stacks with a 7 µm interval and 2 µm pixel size. Imaging of whole tissues was performed in order to eliminate the possibility of subjective selection and assessment of individual fragments of the observed surface. The same exposure settings were used to take images from every round of experiments.

### 4.6. BioFlux Biofilm Model

BioFlux 1000Z setup (Fluxion Biosciences, San Francisco, CA, USA) with an inverted fluorescence microscope (GmbH, Jena, Germany) was used to generate microfluidic conditions of biofilm growth [23]. Firstly, the channels of the BioFlux 48-well microfluidics plate (Fluxion Biosciences, San Francisco, CA, USA) were filled with RPMI 1640 medium and rinsed with a strong medium flow of (10 dyne/cm^2^) for 10 s. Next, 100 µL of each suspension of both *C. albicans* strains, prepared identically as in the microdilution method for MBEC determination (strains subcultured for 24 h on Sabouraud Dextrose Agar with chloramphenicol at a concentration of 100 mg/L, then suspended in RPMI 1640 medium at a concentration of 1–5 × 10^6^ CFU/mL), were placed into the outlet wells, and the flow of the medium was opened from the outlet to inlet channels using a speed rate of 5 dyne/cm^2^ for 5 s. After this step, fungi were left for 1 h in order to allow them to adhere to the channels’ surface. Then, RPMI 1640 was added to inlet wells up to a final volume of 1 mL, and the flow of 0.5 dyne/cm^2^ was set for 24 h. Mature biofilms of *Candida* obtained by the aforementioned method were then either treated with the two tested lipopeptides or not exposed to any antimicrobial compound (a positive control). USCLs with a volume of 1 mL and concentrations equal to the MBECs determined in the previous study [26] were added to the inlet wells, and the flow of 0.5 dyne/cm^2^ was maintained for 24 h. The experiment included two positive controls, with biofilm of the tested fungi in RPMI 1640 not exposed to any compound. For all tested samples, the biofilm obtained after 24 h of growth in the absence of any substances (a biofilm development phase) was taken as the starting point, and the biofilm increase obtained during the second day of biofilm growth was regarded as final result. A time-lapse series of images was taken every 1 h during the entire experiment. The photos obtained in that manner were then analyzed by the BioFlux Montage software. All experiments were repeated in triplicate.

### 4.7. Statistical Analysis

Statistical analysis was performed using the GraphPad Prism version 9 (GraphPad Co., San Diego, CA, USA). The normality of distribution was checked by the Shapiro–Wilk test. As all values were normally distributed, the one-way ANOVA test was further used. The results of statistical analyses were considered significant for values with *p* < 0.05.

## 5. Conclusions

The results presented in our study contradict the results obtained previously during the in vitro study of the efficacy of C_16_-KKKK-NH_2_ and C_16_-CKKKKC-NH_2_ ultrashort lipopeptides against *Candida* strains isolated from VVC. This is because in no case was it possible to achieve a complete eradication of the biofilm. Both the ex vivo animal model using the murine vaginal epithelium and the microfluidic biofilm model failed to confirm the eradicating effects of L1 and C1 at previously determined concentrations (MBEC). In the method using BioFlux, it is possible to observe some weak effect of limiting the further development of fungal biofilm, which is stronger with the use of a linear analog than a cyclic one. In addition, the action of both USCLs at sub-inhibitory concentrations (1/2 MBEC) ex vivo indicates a potentially more beneficial effect of L1, possibly through active sensitization of *Candida* to the action of substances belonging to, for example, HDPs present in the vagina. However, the interactions in the vaginal microenvironment between lipopeptides and possible HDPs require further detailed research focused on identifying such compounds and their mechanisms of action. As in our previous work, the most favorable results were obtained in an ex vivo model when using the combination of USCLs with fluconazole conventionally used in VVC. The use of concentrations corresponding to the lowest previously determined FIC—i.e., many times lower than when applied separately to both plankton (MIC) and biofilm (MBEC)—resulted, in almost every case, in eradication at a higher level than at other concentrations of L1 and C1. Thus, the advantage of combination therapy using compounds with different mechanisms of action over the use of test substances separately was demonstrated once again. It should be emphasized that routine in vitro susceptibility testing of fungi in clinical practice almost always indicates the susceptibility of fungi grown in VVC. In our study, we proved that these methods most likely do not reflect the behavior of *Candida* in vivo during infection, and that the actual eradication of these microorganisms is much more difficult than it would appear from the mycogram. This is most likely the reason for the frequently observed therapeutic failures and for the increasing rate of recurrence of infections.

## Figures and Tables

**Table 1:** Concentration values (µg/mL) for both investigated *Candida* strains (CA1 and CA2) for all tested compounds used in this study: lipopeptides L1 (linear), C1 (cyclic), and fluconazole (FLC), determined in our previous study [26], and amphotericin B (AMB). MIC—minimal inhibitory concentration, MBEC—minimal biofilm eradication concentration, FIC—fractional inhibitory concentration.

	AMB	MIC			MBEC			FIC			
		FLC	L1	C1	FLC	L1	C1	FLC + L1	Interpretation	FLC + C1	Interpretation
**CA1**	50	≤0.125	32	4	>512	256	64	0.001953 + 16	0.875 additive effect	0.0625 + 2	1.0 additive effect
**CA2**	50	≤0.125	32	4	>512	256	64	0.001953 + 8	0.266 synergistic effect	0.0315 + 2	0.75 additive effect

## Data Availability

The datasets generated during and/or analyzed during the current study are available from the corresponding author upon reasonable request.

## Supplementary Materials

The following supporting information can be downloaded at: https://www.mdpi.com/article/10.3390/ijms232214453/s1; Videos S1 and S2 provide animations of all time-lapse microscopic shots captured during the 24 h of incubation of strain CA1 and CA2, respectively, in microfluidic conditions obtained by the BioFlux model. Figures S1–S4 show results of *Candida* biofilm eradication (CFU/g) under the influence of tested substances with statistical analysis, for both strains CA1 and CA2 separately.

## Abbreviations

CA1–*C. albicans* strain no 1	VVC–vulvovaginal candidiasis
CA2–*C. albicans* strain no 2	CFU–colony forming unit
L1–linear analogue 1 (C_16_-KKKK-NH_2_)	MIC–minimum inhibitory concentration
C1–cyclic analogue 1 (C_16_-CKKKKC-NH_2_)	MBEC–minimum biofilm eradication concentration
AMB–amphotericin B	
FLC–fluconazole	FIC–fractional inhibitory concentration
K-–negative control	K–lysine
K+–positive control	R–arginine
USCL–ultrashort cyclic lipopeptides	C16–hexadecenoic acid
AMPs–antimicrobial peptides	NH2–amide residue
HDPs–host defense peptides	VECs–vaginal epithelial cells
