# Supplementary material for: Activity of Novel Ultrashort Cyclic Lipopeptides against Biofilm of Candida albicans Isolated from VVC in the Ex Vivo Animal Vaginal Model and BioFlux Biofilm Model—A Pilot Study
Source: Int J Mol Sci. 2022 Nov 21;23(22):14453. doi: 10.3390/ijms232214453 (PMC9694474; doi:10.3390/ijms232214453)
Supplement: Supplementary file 1 [file ijms-23-14453-s001.zip › Supplementary Materials Figures S1-S4.pdf]

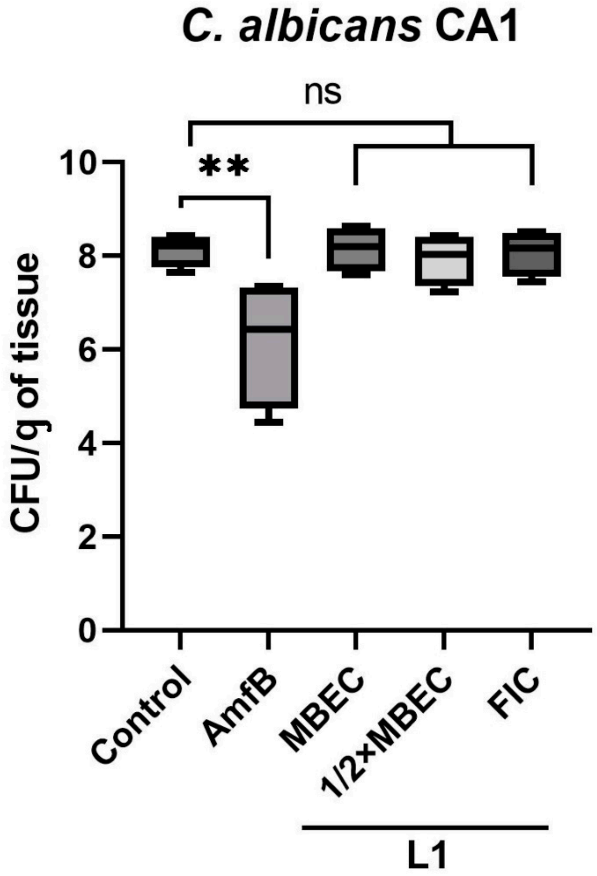

Figure S1. Distribution of CFU per gram of tissue values obtained for strain CA1. Control is referred to as a positive control (untreated biofilm of *C. albicans*). Biofilms were treated with: AmfB – amphotericin B (50 μg/mL), lipopeptide L1 at a concentration equal to MBEC, ½ MBEC and FIC values (MBEC – minimum biofilm eradication concentration; FIC – fractional inhibitory concentration). NS – non significant results (p>0.05), */** - results with statistical significance (p<0.05).

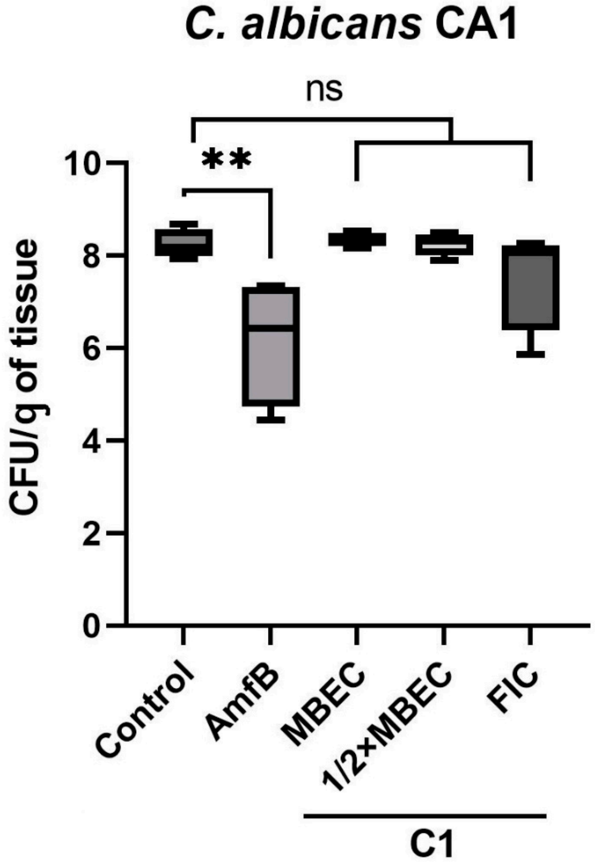

Figure S2. Distribution of CFU per gram of tissue values obtained for strain CA1. Control is referred to as a positive control (untreated biofilm of *C. albicans*). Biofilms were treated with: AmfB – amphotericin B (50 μg/mL), lipopeptide C1 at a concentration equal to MBEC, ½ MBEC and FIC values (MBEC – minimum biofilm eradication concentration; FIC – fractional inhibitory concentration). NS – non significant results ($p>0.05$), */** - results with statistical significance ($p<0.05$).

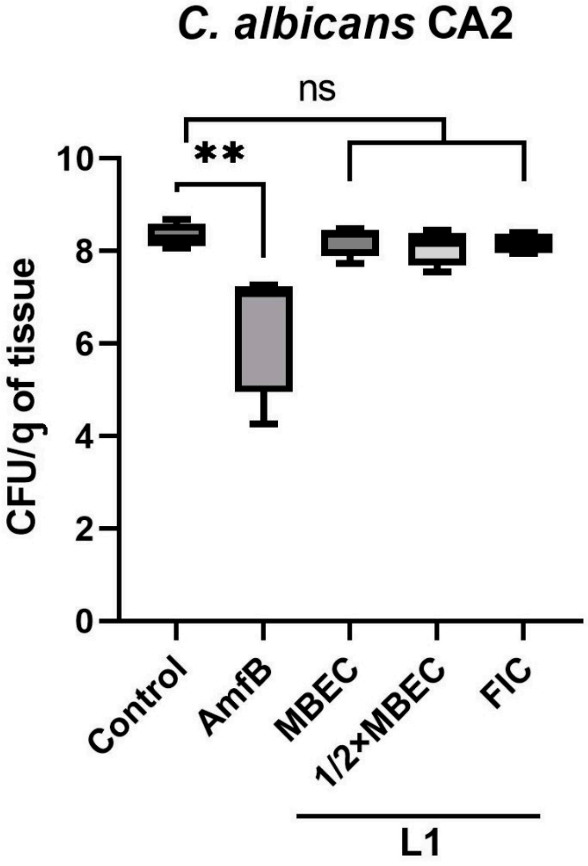

Figure S3. Distribution of CFU per gram of tissue values obtained for strain CA2. Control is referred to as a positive control (untreated biofilm of *C. albicans*). Biofilms were treated with: AmfB – amphotericin B (50 μg/mL), lipopeptide L1 at a concentration equal to MBEC, ½ MBEC and FIC values (MBEC – minimum biofilm eradication concentration; FIC – fractional inhibitory concentration). NS – non significant results (p>0.05), */** - results with statistical significance (p<0.05).

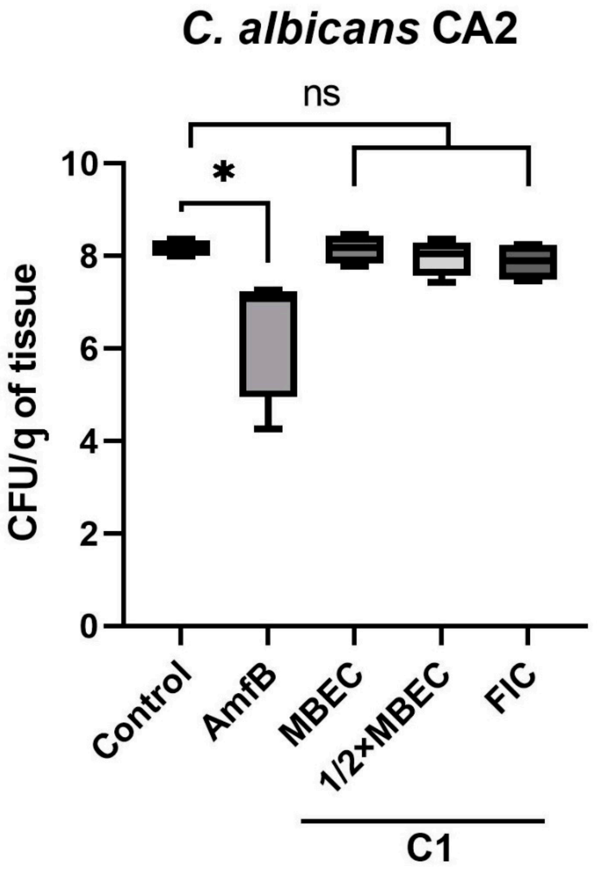

Figure S4. Distribution of CFU per gram of tissue values obtained for strain CA2. Control is referred to as a positive control (untreated biofilm of *C. albicans*). Biofilms were treated with: AmfB – amphotericin B (50 µg/mL), lipopeptide C1 at a concentration equal to MBEC, ½ MBEC and FIC values (MBEC – minimum biofilm eradication concentration; FIC – fractional inhibitory concentration). NS – non significant results ($p>0.05$), */** - results with statistical significance ($p<0.05$).
